# Oxidation reactions of cellular and acellular hemoglobins: Implications for human health

**DOI:** 10.3389/fmedt.2022.1068972

**Published:** 2022-11-28

**Authors:** Abdu I. Alayash

**Affiliations:** Laboratory of Biochemistry and Vascular Biology (LBVB), Center for Biologics Evaluation and Research (CBER), Food and Drug Administration (FDA), Silver Spring, MD, United States

**Keywords:** hemoglobin, oxidation, blood substitutes, stored blood, ferryl heme species

## Abstract

Oxygen reversibly binds to the redox active iron, a transition metal in human Hemoglobin (Hb), which subsequently undergoes oxidation in air. This process is akin to iron rusting in non-biological systems. This results in the formation of non-oxygen carrying methemoglobin (ferric) (Fe^3+^) and reactive oxygen species (ROS). In circulating red blood cells (RBCs), Hb remains largely in the ferrous functional form (HbF^2+^) throughout the RBC's lifespan due to the presence of effective enzymatic and non-enzymatic proteins that keep the levels of metHb to a minimum (1%–3%). In biological systems Hb is viewed as a Fenton reagent where oxidative toxicity is attributed to the formation of a highly reactive hydroxyl radical (OH^•^) generated by the reaction between Hb's iron (Fe^2+^) and hydrogen peroxide (H_2_O_2_). However, recent research on both cellular and acellular Hbs revealed that the protein engages in enzymatic-like activity when challenged with H_2_O_2_, resulting in the formation of a highly reactive ferryl heme (Fe^4+^) that can target other biological molecules before it self-destructs. Accumulating evidence from several *in vitro* and *in vivo* studies are summarized in this review to show that Hb's pseudoperoxidase activity is physiologically more dominant than the Fenton reaction and it plays a pivotal role in the pathophysiology of several blood disorders, storage lesions associated with old blood, and in the toxicity associated with the infusion of Hb-derived oxygen therapeutics.

## Introduction

### Oxidation reactions of hemoglobin

The primary function of Hb within the RBC is to transport oxygen from lungs to tissues and to facilitate the removal of carbon dioxide (CO_2_) that accumulates in tissues due to active metabolism. The molecule is made up of four subunits: two *α* and two *β* (*α* with 141 amino acids and ß with 146 amino acids). The subunits are packed in pairs forming a tetramer. Each globin chain carries a heme prosthetic group surrounded by hydrophobic amino acids. The *α*1ß2/*α*2*β*1 interface plays a crucial role in oxygen binding, as oxygen is released through a subunit rearrangement in the tetramer resulting in signiﬁcant quaternary conformational changes during the binding of oxygen (1).

In biological systems, Hb's iron promotes the generation of ROS which involves a reduction of oxygen (O_2_) by one electron, forming superoxide (O_2_^•−^). This superoxide ion can be dismutated to yield H_2_O_2_ that, on subsequent reduction, forms hydroxyl radicals (OH^•^). This form of Hb oxidation is known as the Fenton reaction (2) and can be seen in Equation 1:

(1) $$Fe^{2+}+H_{2}O_{2}\to Fe^{3+}+OH^{∙}+OH^{-}$$

This hypothesis, however, was questioned as it was pointed out more recently that in the case of the Fenton reaction, H_2_O_2_ reacts directly with the free iron but not with iron that is still part of Hb's prosthetic group (3–5).

In recent years, oxidation reactions of Hb took a center stage as researchers and developers focused on cell-free Hb as a starting material for the manufacture of Hb-based oxygen carrier (HBOC) therapeutics. Proposed alternative pathways describing *in vitro* and *in vivo* Hb oxidation reactions fall into two categories. First, Hb in an oxygenated medium undergoes spontaneous oxidation of its ferrous iron to form the ferric-non oxygen carrying protein. This reaction is referred to as the autoxidation process of Hb and described by Equation 2:

(2) $$\mathrm{HbF}e^{2+}O_{2}\to \mathrm{HbF}e^{3+}+O^{∙-}\;$$

In this reaction an anion-induced autoxidation of Hb occurs in which nucleophilic anion displacement of molecular oxygen results in an intermediate ferrous heme/anion complex that acts as an electron donor to displaced oxygen (6).

In the presence of small quantities of H_2_O_2_, or when H_2_O_2_ is generated enzymatically by systems such as xanthine oxidase or glucose oxidase, Hb enters a vicious redox cycle that generates several damaging oxidative intermediates. This peroxidatic action is often termed as pseudoperoxidase activity. In general, the ﬁrst product of the reaction of ferrous Hb with H_2_O_2_ is an oxo ferryl heme (Equation 3). The reaction of the ferric heme with H_2_O_2_ on the other hand leads to the production of a porphyrin radical cation (the protein radical cation *P*^•+^ deprotonates to yield the stable electron paramagnetic resonance [EPR] detectable *P*^•^) that can be damaging to the Hb itself as it migrates to cysteine-93 of the *β* chain and other “hotspot” amino acid targets (Equation 4) (7).


(3) $$\mathrm{HbF}e^{2+}+H_{2}O_{2}\to \mathrm{HbF}e^{4+}=O^{2-}+H_{2}O\;$$

(4) $$\mathrm{HbF}e^{3+}+H_{2}O_{2}\to P^{∙+}\mathrm{HbF}e^{4+}=O^{2-}+H_{2}O\;$$

In addition, a covalent heme-to-protein link has been detected by HPLC during the reaction of Hb and H_2_O_2_. This adduct has been recognized as a valuable biomarker for the peroxidatic activity of myoglobin and Hb (7, 8).

## Oxidation reactions of acellular hemoglobin: the case of hemoglobin-based oxygen carriers

### HBOCs manufacturing and developments

HBOCs have been developed as oxygen carrying solutions for a variety of clinical applications, most typically as a substitute for allogeneic RBC transfusion in settings where transfusion is indicated, but RBC are unavailable. This includes cases of major hemorrhage, whether traumatic or surgically induced; where compatible blood is unavailable; or where transfusion therapy is refused on religious grounds. Potential benefits include universal compatibility, immediate availability, and long-term storage. However, no HBOCs have been approved by the US FDA as if yet (9). Oxyglobin was approved by US FDA in 1997 for veterinary use only and in 1998 was approved in Europe.

Over the last three decades there has been an intense research and development effort in using acellular Hb as an oxygen carrying therapeutic in transfusion medicine. This led to increasing opportunities for researchers to work on Hb oxidation reactions in this unique and challenging environment, which led to the unraveling of several novel oxidative pathways (10).

HBOCs are derived from outdated human blood or in some cases from animal blood after extensive purification and filtration processes. The cell-free Hb, also known as stroma free Hb is used as a starting material for further processing to ensure the complete removal of RBC proteins and enzymes. In some cases, manufacturers applied further chromatographic procedures to produce a highly purified HbA (known as HbA_0_) as starting material for subsequent chemical modifications (11). Chemical modifications have been widely used to generate variable size HBOCs, using polymerizing reagents such as glutaraldehyde and polyethylene glycol which result in inter- and intramolecular crosslinked stabilized tetramers or conjugated/polymerized molecules. These modifications are non-site specific and, in some cases, random modifications of the Hb molecule occur (12).

Genetically modified HBOCs have also been expressed in *E. coli* and have advanced to late-stage clinical trials in the case of some chemically modified Hbs. Because of the potential side effects of free Hb, crosslinking of Hb with antioxidative enzymes and/or encapsulation of the protein inside lipid vesicles with antioxidative enzymes have been investigated as an alternative model system mimicking RBCs (13, 14). Second-generation versions of these vesicles are now under active investigation (15).

Several newly developed third generation HBOCs, such as HEMO2life has recently been approved in Europe for kidney transplant donor perfusion. This is an extracellular Hb derived from see worms and it is a multicomponent molecule carrying multiheme sites with a large oxygen-binding capacity (16). OxyVita is another newly developed HBOC produced by using a modified zero-linked polymerization process that employs chemical activators to incorporate cross-linked bovine Hb tetramers into “super-polymeric” macromolecules (17).

A first-in-human Phase 1 clinical trial assessing the safety and pharmacokinetics of Hb vesicles (HbV) in healthy male adult volunteers was recently reported from Japan (18). HbV infusion (with half-life of approximately 8 h) was well tolerated and all adverse events observed, including liposome-induced infusion reactions, were spontaneously resolved. Dose escalation studies are planned for a larger number of subjects in a Phase 2 clinical trial.

Because of the proprietary nature of the commercially manufactured HBOCs, it was not until recently that a very comprehensive comparative study of all HBOCs (tested in humans in late clinical trials) was published (19). Generally, HBOCs were found to be variable in their autoxidation rates, oxidative changes, and heme loss kinetics, in large part due to the varied and random nature of chemical modifications. When these HBOCs were challenged with H_2_O_2_, some underwent oxidative changes that were more exaggerated than others, leading to the accumulation of higher levels of oxidation intermediates (ferryl in particular) compared to the unmodiﬁed human or bovine forms (19).

### Invitro and *in vivo* cardiovascular effects of HBOCs with a focus on oxidative pathways

Cardiovascular effects were one of the most frequently reported adverse events experienced by human volunteers infused with HBOCs. The development of cardiac lesions after infusion of some HBOCs in several animal models was used to illustrate the potential role of oxidative pathways in development of lesions in the heart. Myocardial lesions following administration of DCLHb in several animal models have been reported (20).

Due to their relatively long circulation time, acellular Hbs are exposed to oxidative changes and metHb accumulation. For example, rapid oxidation of some HBOCs in circulation has been reported reaching levels as high as 40%–60% metHb in humans after transfusion (21).

An isolated rat heart Langendorff perfusion system was recently used to monitor the recovery of left ventricular functions following hypoxic perfusion with ferrous and ferric forms of diaspirin crosslinked human Hb (DCLHb [also known as DBBF] developed by Baxter). Morphological and biochemical changes, including the development of heart lesions, were documented and changes in tissue marker oxidation (i.e., lipid peroxidation and heme oxygenase expression) were also observed. At the subcellular levels, ferric and possibly ferryl Hb induced impairment of mitochondrial function associated with the inhibition of state 3 respiration and cytochrome c oxidase activity as well as changes in the heart tissue proteome. Co-perfusion of hearts with ferrous DCLHb/DBBF and ascorbic acid (Asc) under normoxia led to a sharp decline in cardiac parameters. This trend continued with ferric Hb co-perfusion, but only at the higher concentration of Asc (22).

Hemodynamic changes in humans due to the scavenging of the blood vessel vasodilator nitric oxide (NO) became a serious impediment to the progress and clinical utility of HBOCs (23, 24). NO reacts avidly with infused HBOCs regardless of their molecular sizes or shapes, resulting in blood pressure elevation due to the vasoconstriction of blood vessels. Sodium nitrite (NaNO_2_) was used as a mechanism to replace scavenged NO. One lesser-known attribute of HBOCs is that they exhibit some nitrite reductase activity; therefore, Hb can become a source of NO in the presence of NaNO_2_ (24).

In a swine model of liver hemorrhage, Biopure's HBOC-201 was infused with or without concurrent NaNO_2_ and moderation of vasoconstriction was indeed achieved. However, the highest incidence of adverse events, including pulmonary complications, were also recorded in a dose-dependent fashion (25). In a guinea pig model, known for its inability to synthesize Asc, infusion of nitrite with an HBOC (DCLHb/DBBF) potentiated renal oxidative stress and injury in these animals with large accumulation of metHb (26). Although no attempts were made to look at the ferryl fingerprints, nitrite is known to directly interact with *β*Cys93 which may destabilize Hb (27).

To establish a role for Asc as an effective endogenous reducer of Hb oxidation intermediates, a glutaraldehyde polymerized bovine Hb, Oxyglobin™ (Biopure), was infused to rats and guinea pigs. Rats can endogenously synthesize Asc, but guinea pigs, like humans, lack the enzymatic machinery to produce it. This experiment demonstrated clearly that the ferric HBOC levels were 4-fold greater in the guinea pig compared to the rat. HPLC and mass spectrometric methods also showed oxidative instability of Oxyglobin^™^ following administration in the guinea pig but not in the rat (28).

To capture ferryl/ferryl radicals or endogenous defenses invoked against them such as plasma Asc, EPR was used in a rabbit model of 20% blood for HBOC (DCLHb/DBBF) exchange transfusion. Rabbits, unlike humans, maintain an effective Asc reducing system in their blood. In these experiments, it was noted that metHb levels in circulation were reduced to oxyHb by a slow process (t_1/2_=1 h), with no globin-bound free radicals found in the plasma of these animals (29). It was noted, however, that endogenous Asc was able to effectively reduce plasma metHb, ferrylHb, and its associated globin radicals. The detection of ascorbyl free radicals by EPR was used to confirm that the intraerythrocytic Asc acted as the electron donor (29, 30).

Another study in humans that confirmed the role of Asc in controlling Hb oxidation involved a Jehovah Witness patient who lost a considerable amount of blood. This patient was given large doses of Asc after transfusion with the human analog of Oxyglobin, Hemopure, which led to a considerable reduction in this patient's oxidized Hb levels (21). Therefore, in severe anemia or when HBOCs are infused antioxidants, such as ascorbic acid may be indicated as exogenous agents to control methemoglobinemia.

## Oxidation reactions of cellular hemoglobin in health and in disease states

### Oxidation reactions in Normal red blood cells

RBCs maintain very effective antioxidative enzyme systems that keep Hb in the ferrous functional form in circulation during its lifespan. However, as cells age, oxidation of Hb and oxidative side reactions intensify due to the weakening of these antioxidative defense mechanisms (31). The antioxidative defense enzymes in normal RBCs include superoxide dismutase, catalase, glutathione peroxidase, and glutathione reductase. Other low-molecular-weight antioxidants, such as glutathione, and vitamins E and C are also known to contribute to the overall control of Hb oxidation within cells. RBCs also maintain a plasma membrane redox system that transfers electrons from intracellular substrates to extracellular electron acceptors, which may be NAD+or vitamin C (32). Therefore, RBCs are uniquely designed to transport oxygen as well as providing enzymatic mechanisms to maintain Hb in a functional nontoxic state (33).

Band 3 and its associated proteins are an integral part of the RBC membrane and are responsible for maintaining acid balance, ion distribution (Cl^−^ and HCO3^−^) across the RBC membrane exchange, and cell shape integrity and durability (34). More recently a role for band 3 in the overall maintenance of the redox balance in RBCs has been identified (see below).

### Pathophysiology of oxidation reactions and the detection of ferryl hemoglobin *in vivo*

Recent experiments from our laboratory on blood from transgenic sickle cell disease (SCD) mice and from patients with SCD showed that Hb's higher oxidation state, ferrylHb, interacts directly with band 3 resulting in oxidative modifications of the band 3 network of proteins (35, 36). These experiments also confirmed that Hb mediated oxidation of band 3 triggers band 3 clustering and microparticle (MP) formation and release from mother cells. MPs are miniature RBCs containing almost all the elements of mature RBCs, including Hb (for review, see 37).

Photometric and proteomic analyses of the MP content from a Townes-sickle cell mice model showed the presence of considerable levels of Hb oxidation intermediates (ferric/ferryl). Moreover, it was also shown by mass spectrometry in these experiments that the degree of *β*-globin post-translational modiﬁcations (PTMs) in Hb within MPs, including irreversible oxidation of *β*Cys93 and the ubiquitination of *β*Lys96 and *β*Lys145 (35), was very evident. A definitive follow-up experiment also revealed that ferryl Hb, but not hemichromes, was found to induce the complex formation with band 3 and RBC membrane proteins, consistent with early *in vitro* reports (38). When MPs obtained from Townes-SS mice that were fed a diet rich in hydroxyurea (HU), fewer PTM modiﬁcations were found on the Hb. *In vitro*, HU (an NO producing molecule) reduced the levels of ferryl Hb and shielded its target residue, *β*Cys93, by a process of S-nitrosylation (35).

In a follow up clinical investigation using MPs from blood of SCD patients who were either on or off HU treatment was carried out recently (36). Proteomic analysis showed that band 3 and its interaction network were involved in MP formation. Samples from SS patients exhibited more extensive protein phosphorylation and ubiquitination in SCD patients than in samples from ethnic matched controls. Samples from patients who were treated with HU showed little or no oxidative PTMs like those samples from the control group (36).

Several studies in recent years focused on detecting the short-lived ferryl and its radical in human blood/tissues and animal models. An EPR study (39) detected a protein based radical at ∼1 *μ*M concentration in human venous blood samples. This radical was identical in line-shape and power saturation characteristics to that generated from *in vitro* experiments through the reaction of ferric Hb with H_2_O_2_. In addition, characteristic EPR protein radical signal was detected, representing oxidative damage to the heme and by implication the oxoferryl species. Similar ferryl mediated-protein-heme modiﬁcations were reported in acute kidney dysfunction following rhabdomyolysis (8) and in cerebral spinal ﬂuid following subarachnoid hemorrhage (40). Oxidized Hb intermediates and cross-linked globin chains were confirmed by another group using LC-MS/MS in atherosclerotic lesions of the human carotid artery and hemorrhagic cerebrospinal ﬂuid from preterm infants (41). In a follow-up study, the same group used speciﬁc monoclonal anti-ferrylHb antibody and found that ferrylHb was localized extracellularly and internalized by macrophages in the human hemorrhagic complicated lesions (42).

Hb's pseudoperoxidase activity is best illustrated in sickle cell disease, a biologically more relevant model system for the investigation of Hb oxidation reactions in humans and animals. Sickle cell Hb (HbS) oxidizes faster and its ferryl persists longer in solutions than ferryl HbA (43). *In vivo* verification of the ferryl heme was therefore achievable. One of the early reports on the persistence of ferrylHb in SS RBCs infected with malarial parasites found that ferryl Hb inhibited actin polymerization in RBCs-infected malaria, thereby preventing the malarial parasites from creating their own actin cytoskeleton within the host cell cytoplasm (44, 45). This also shed some light on the question of how HbS provides protection against malarial parasitic infection in SCD (46).

Pseudoperoxidative activity of Hb and its potential impact on the integrity and long-term stability of blood stored in cold temperatures for 42 days was recently discussed (47). These reactions are collectively known as “oxidative lesion” which may impact clinical outcomes associated with the use of stored blood or pathogen inactivated blood for therapeutic purposes (47).

Under standard storage conditions, Hb oxidation is seen due to a decrease in its antioxidant capacity. This results in the oxidation and deterioration of membrane lipids and proteins, which can ultimately lead to irreversible damage to the membrane.

Most used commercial methods for pathogen reduction and/or inactivation processes are largely based on exposing major blood components (RBCs, platelets, and plasma) to UV light and photosensitizer molecules. UV light radiation of blood contained in plastic bags may lead to the deterioration of RBCs (48). The participation of ROS may drive some of the observed biochemical changes that included cytosolic proteins (including Hb) and lipid proteins (49). Although very little experimental evidence is available that points to the involvement of Hb's pseudoperoxidase activity, storage conditions or inactivation processes under some circumstances may favor these reactions.

## Summary and conclusion

Under oxidative stress conditions, the redox active protein Hb triggers a pseudoenzymatic cycle within RBCs. Outside the RBCs, such as in the case of Hb-based oxygen therapeutics or hemolytic conditions, this effect is amplified. The byproduct of this reaction and most active intermediate, ferryl Hb, has been detected in several *ex-vivo* and *in vivo* model systems, in atherosclerotic lesions of carotid arteries, in blood from mice and SCD patients, and in blood from SCD patients infected with malaria. Unique cellular, and in some instances subcellular, injuries have been attributed to the ferryl Hb's redox reactivity. These reactions are also suspected to be responsible for the oxidation lesions associated with stored blood or pathogen-inactived blood. The knowledge gained in the understanding of the underlying mechanisms of Hb oxidative pathways led to the rational design of several protective intervention strategies that will hopefully augment the safe use of blood for therapeutic purposes.
